# EOTE-FSC: An efficient offloaded task execution for fog enabled smart cities

**DOI:** 10.1371/journal.pone.0298363

**Published:** 2024-04-05

**Authors:** Faheem Nawaz Tareen, Ahmad Naseem Alvi, Badr Alsamani, Mohammed Alkhathami, Deafallah Alsadie, Norah Alosaimi

**Affiliations:** 1 Department of Electrical and Computer Engineering, COMSATS University Islamabad, Islamabad, Pakistan; 2 Information Systems Department, College of Computer and Information Sciences, Imam Mohammad Ibn Saud Islamic University (IMSIU), Riyadh, Saudi Arabia; 3 Information Systems Department, Umm Al-Qura University, Mecca, Saudi Arabia; University of the West of Scotland, UNITED KINGDOM

## Abstract

Smart cities provide ease in lifestyle to their community members with the help of Information and Communication Technology (ICT). It provides better water, waste and energy management, enhances the security and safety of its citizens and offers better health facilities. Most of these applications are based on IoT-based sensor networks, that are deployed in different areas of applications according to their demand. Due to limited processing capabilities, sensor nodes cannot process multiple tasks simultaneously and need to offload some of their tasks to remotely placed cloud servers, which may cause delays. To reduce the delay, computing nodes are placed in different vicinitys acting as fog-computing nodes are used, to execute the offloaded tasks. It has been observed that the offloaded tasks are not uniformly received by fog computing nodes and some fog nodes may receive more tasks as some may receive less number of tasks. This may cause an increase in overall task execution time. Furthermore, these tasks comprise different priority levels and must be executed before their deadline. In this work, an Efficient Offloaded Task Execution for Fog enabled Smart cities (*EOTE* − *FSC*) is proposed. *EOTE* − *FSC* proposes a load balancing mechanism by modifying the greedy algorithm to efficiently distribute the offloaded tasks to its attached fog nodes to reduce the overall task execution time. This results in the successful execution of most of the tasks within their deadline. In addition, *EOTE* − *FSC* modifies the task sequencing with a deadline algorithm for the fog node to optimally execute the offloaded tasks in such a way that most of the high-priority tasks are entertained. The load balancing results of *EOTE* − *FSC* are compared with state-of-the-art well-known Round Robin, Greedy, Round Robin with longest job first, and Round Robin with shortest job first algorithms. However, fog computing results of *EOTE* − *FSC* are compared with the First Come First Serve algorithm. The results show that the *EOTE* − *FSC* effectively offloaded the tasks on fog nodes and the maximum load on the fog computing nodes is reduced up to 29%, 27.3%, 23%, and 24.4% as compared to Round Robin, Greedy, Round Robin with LJF and Round Robin with SJF algorithms respectively. However, task execution in the proposed *EOTE* − *FSC* executes a maximum number of offloaded high-priority tasks as compared to the FCFS algorithm within the same computing capacity of fog nodes.

## Introduction

Smart cities are currently in high demand, not only in emerging regions but also in well-established urban areas. Their appeal lies in the enhanced quality of life they offer to their residents. These cities heavily rely on Information and Communication Technologies (ICT) to manage crucial aspects of urban infrastructure, such as water, waste, and energy, as well as to bolster health, safety, and security [1–5].

Data collection within smart cities is carried out through a network of sensors situated in infrastructure, buildings, and assets. This data is subsequently analyzed to automate services, optimize performance, reduce operational costs, and make the best use of limited resources. It serves a multitude of purposes, from managing power plants, water supply, and transportation, to enhancing public services like education and healthcare, and even law enforcement and emergency response.

**Fig 1:**
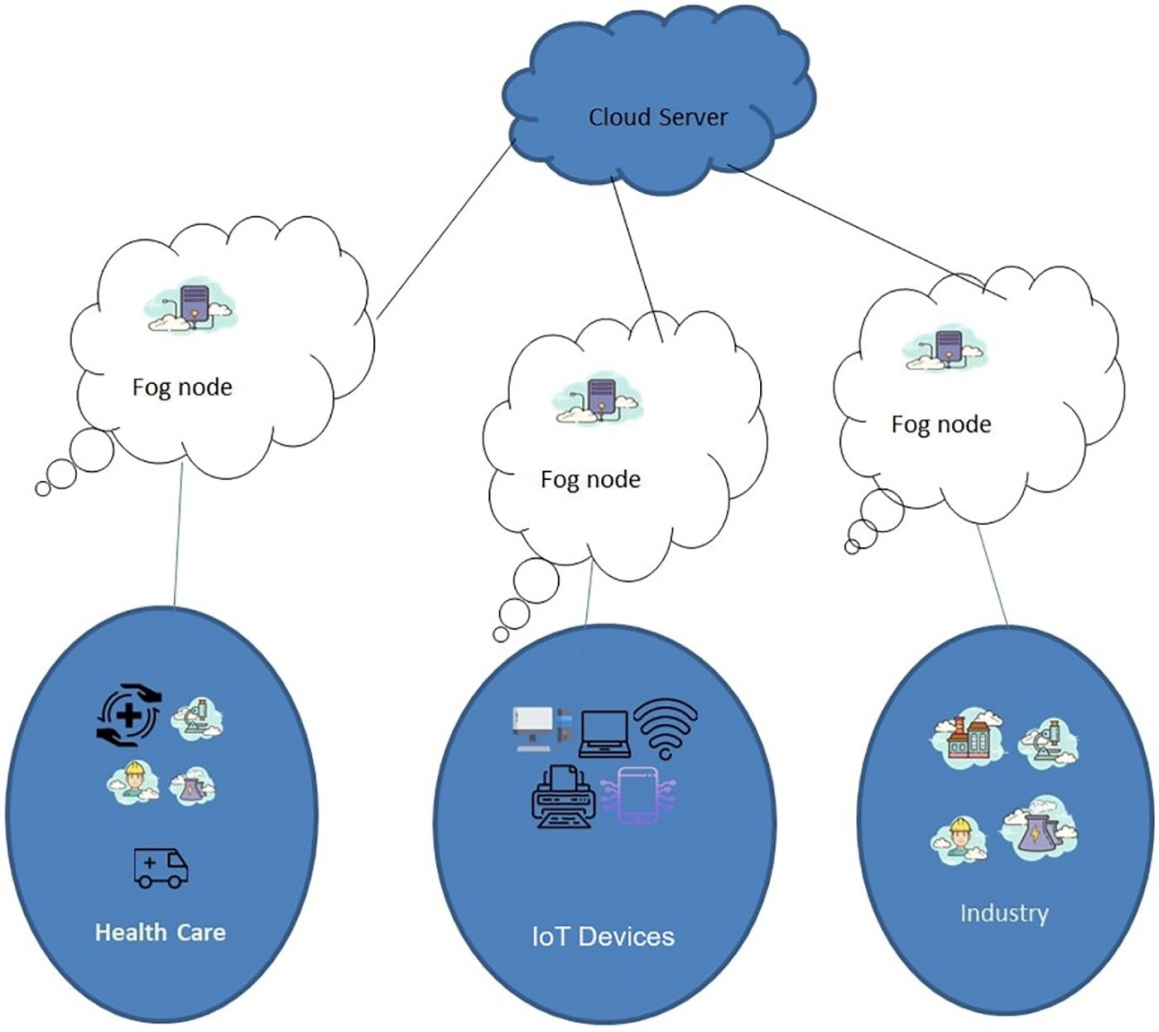
Smart city connectivity with fog.

Key technologies that underpin smart cities include artificial intelligence, communication networks, cloud computing, mesh networks, and the Internet of Things (IoT) [6, 7]. These technologies facilitate data exchange among various connected devices, where data collected by IoT sensors deployed across various smart city applications is stored in a centralized database, typically located in cloud servers. Cloud servers offer advantages such as substantial storage capacity and computational capabilities, which result in more cost-effective data management. However, at the same time, due to its remote placement, propagation delay increases resulting in an increased latency. This limitation can be overcome by placing machines in close vicinity of IoT nodes in different locations of smart city applications such as “fog computing” [8, 9]. Fog computing nodes are accessible within a single network hop and serve as intermediaries between remote cloud servers and end-users, thus reducing latency. Fog nodes are mostly accessible to users on a single hop and act as a bridge between cloud servers and end-users as shown in Fig 1.

Fog computing is an architectural concept characterized by the placement of one or more data centres at the periphery of user networks, rather than routing data over the internet backbone [10–13]. This setup, existing within the same IoT network, allows various sensor devices to transmit their data swiftly for expedited processing. Fog computing employs intelligent sensing to reduce the burden on cloud capacity by filtering out irrelevant data. Various data synchronization techniques, such as Push schemes, Pull schemes and Push-Pull schemes, are employed to collect data from lower-end fog nodes to higher-end fog nodes for manageable processing. These schemes adhere to communication protocols tailored to the fog computing environment.

Fog nodes store and process data at the edge of devices, whereas cloud computing relies on centralized data centres accessed through the internet. This proximity allows fog nodes to swiftly process tasks, making them the preferred choice for applications that require minimal delays. Due to low latency, fog computing is widely acceptable for time-constraint applications in urban management [14, 15].

Wireless sensor-based IoT nodes in smart cities have limited computing and processing capabilities and executing complex tasks takes more time, which is sometimes not required in time-constrained applications. To execute these tasks in a specific time frame, these tasks are required to be offloaded to high processing and computing machines in the close vicinity of these IoT nodes, such as fog computing nodes.

These offloaded tasks are different and it is quite possible that some of the fog nodes receive less number of tasks and some may experience a large number of offloaded tasks. This creates an imbalance load on fog nodes resulting in overall increased execution time. To reduce this execution time, offloaded tasks are required to be uniformly distributed among all fog nodes. This can be achieved by placing a load-balancing node between the IoT nodes and fog computing nodes.

There are different types of load balancing schemes used in allocating the tasks among computing nodes. Some of the most commonly used allocation schemes are Random allocation, Round Robin, Round Robin with LJF, and Greedy algorithm.

In a random algorithm, the load balancer allocates the tasks randomly. It is easy to deploy, however, its latency issues are very high. The latency issues have been somehow addressed in Round Robin by allocating the tasks in a round-robin fashion. However, it does not consider the size and complexity level of the tasks. Due to this, the tasks are not uniformly distributed. Round Robin with LJF proposed in [16] overcome this issue somehow. However, there are quite several task patterns that are not uniformly allocated. A greedy algorithm allocates the tasks to machines by considering the load on the machines and assigns the tasks to such machine that has the least processing capacity.

Though all the above-mentioned schemes, reduce the task execution time, however, there is still room to improve the execution time of these tasks. In this work, an Efficient Offloaded Tasks Execution scheme for Fog enabled Smart Cities (*EOTE* − *FSC*) is proposed. The salient features of *EOTE* − *FSC* are mentioned below.

1. Introduce a hierarchical system model with a load balancer for fog-enabled smart city applications.
2. A load balancing algorithm for load balancers to uniformly distribute the offloaded tasks among fog computing nodes to reduce the execution time.
3. An algorithm for a fog computing node to efficiently execute the offloaded tasks in such a way that the majority of the offloaded tasks are executed to meet their deadline.

The rest of the paper is organized as: At first, the research work related to task offloading particularly in fog computing is discussed. This is followed by the proposed scheme with its different algorithms. The system model along with the comparative results are analyzed afterwards. Finally, conclusions are provided at the end.

## Related research

Fog computing is under hot research because it offers reduced delay with immediate processing capabilities for many of the delay-sensitive offloaded tasks.

In [17], the authors highlighted the limited computing resources for the most critical and sensitive tasks in vehicular networks. The authors propose a solution for vehicular fog computing with the help of mobile devices. The roadside Unit (RSU) performs task scheduling among all the vehicles by applying greedy scheduling algorithm. This algorithm was proposed to optimize the latency in the offloaded task. Lastly, the author describes some future work regarding task optimization and load balancing.

In [18], the author presented a meta-heuristic model inspired by nature and solved the latency issues by using fog computing. The proposed model is compared with different scheduling algorithms, including round-robin, throttled algorithm for scheduler and bee fly algorithm. The proposed algorithm only takes 106.5 milliseconds to process. Compared to all other algorithms, it takes less time but does not consider power consumption. They only consider the latency processing time.

In [19], authors proposed a task-offloading scheme for an edge computing network of vehicles by optimizing the selection of fog nodes. The load balancing technique is proposed for offloaded task executions that include two theory-based game schemes for selecting fog nodes and offloaded task decisions with software-defined networking. Authors claim that their proposed scheme reduces the total processing delay. The system design proposed in [20] is based on cloud and fog computing. The authors applied the shortest job first algorithm in assigning tasks on different machines for load balancing. The author compares their result with the round-robin algorithm. The main disadvantages of this paper are Low performance and the Processes having a more waiting time.

In [21], the authors describe the offloading task, specifically decision problems in several users and the structured task circumstances. In edge computing, if the user has a large number of tasks, then it can offload their task for lower energy consumption. There are many offload points but limited resources. In this paper, the author designed the offloading model that has a large number of users and many offloading points. Every user has a well-structured task and has a limited offloading point. Then they apply the backtracking in the offloading decision for the exact solution. Two methods are designed to overcome the time latency: the greedy strategy and the genetic algorithm. Finally, for the simulation, they compare the entire algorithm’s result. The result of the comparison suggests that the greedy algorithm is the best. It reduces the total cost to 55%.

In [22] proposed a Fog-Cloud system that uses a Stochastic Gradient Descent (SGD) algorithm and provided a mathematical model. The authors claimed that their proposed scheme allows smart devices to use less energy, work faster, and cost less when getting help from faraway servers. In [23], authors addressed scalability issues with the increased number of devices and proposed a distributed load-sharing MEC network with cloud cooperation. The authors optimized offloading probability and transmission power by applying queuing theory and SGD algorithm. The authors claimed that their proposed scheme is effective for scalability and energy consumption and handles computation offloading challenges for smart devices. In [24], the authors proposed a scheme to handle heavy tasks by proposing a Mobile Cloud Computing (MCC). The authors claimed that sharing the workload with the cloud to make things faster and save energy.

There are multiple frameworks proposed for the deployment in different offloading scenarios [25–27]. In [25], authors proposed a machine learning-based Context-Sensitive Computational Offloading System (CSOS). CSOS trained on context database and tested on previously selected four algorithms. Authors claimed that CSOS provides high accuracy in offloading decisions. In [26], authors proposed a framework for Mobile Code Offloading for IoT devices called MobiCOP-IoT, which allows to deployment of multiple surrogates on clouds as well as on edge nodes. Authors claimed that MobiCOP-IoT enhances computing capabilities by taking advantage of mobile edge computing by testing it in different scenarios. In [27], authors designed an Evidence-based Mobile Computational Offloading (EMCO) toolkit as a novel solution for computational offloading. The toolkit was tested by deploying it in Amazon EC2 Ireland and claimed that their designed toolkit improves the app execution with reduced energy. In addition, the authors claimed that EMCO offers scalability without compromising its performance.

A comparative summary of all the discussed research articles is shown in Table 1.

**Table 1:** Comparison table of related work.

*Ref. No*.	*The goal of the technique*	*Key Idea*	*Results*
[17]	latency optimization in vehicular network	Mobile devices and Greedy scheduling algorithm	reduced delay
[18]	Reducing processing time	meta-heuristic model without considering power consumption	processing time reduced from round-robin and bee fly algorithms
[19]	load balancing based task offloading	Two theory-based game schemes are proposed	Reduced task-processing time
[20]	load balancing for fog and cloud	Shortest Job First algorithm for load balancing	reduces execution time as compared to round-robin
[21]	Offloading task execution	greedy and genetic algorithms are proposed	reduces the total cost to 55%
[22]	Offloaded tasks for fog-cloud system	Stochastic Gradient Descent algorithm with mathematical modelling	Energy minimized with less cost
[23]	Addresses scalability issues with increased number of devices	Queuing theory with Stochastic Gradient Descent algorithm	Offers scalability with reduced energy consumption
[24]	Addresses heavy offloaded tasks	Mobile cloud computing for offloaded tasks on cloud	Fast processing with energy-saving
[25]	Trained context database	Machine learning-based context-sensitive computation	High accuracy in offloading decisions
[26]	Framework for offloading mobile codes in IoT	Deployment of multiple surrogates on cloud	Enhance computing capabilities
[27]	A toolkit for evidence-based mobile computational offloading	Deployed the toolkit in Amazon EC2	Improves app execution with reduced energy

## Proposed scheme

In the proposed *EOTE* − *FSC*, sensor nodes are deployed in different locations for different smart city applications. These sensor nodes are connected with one of the sparsely located fog nodes. IoT sensor nodes have diverse nature of tasks with different execution times. Due to low processing capacity, IoT nodes are unable to execute these tasks within the required time frame. In such cases, complex tasks are offloaded to fog nodes that are placed in close vicinity of these nodes. Due to the diverse nature of these tasks, it is quite possible that some fog nodes are heavily loaded and some are lightly loaded or have no tasks to execute. This results in an unbalanced load on fog nodes resulting in an overall increase in their task execution time. To minimize the overall execution of time, these tasks are required to be uniformly distributed among fog nodes. The main purpose of the *EOTE* − *FSC* scheme is to reduce the overall task execution time of all offloaded tasks by proposing the concept of a load balancer along with the following two algorithms.

1. One algorithm is proposed for load balancing machines to efficiently distribute the offloaded tasks to its directly connected fog node.
2. The Second algorithm is for the fog node to execute tasks according to their priorities and their deadline.

IoT devices continuously send their data to the fog node. Fog nodes are deployed in different locations of smart cities with limited processing capability. Fog nodes are placed in different locations in the smart city. Fog nodes do not receive a uniform number of offloaded tasks from sensor nodes, resulting in an off-balance situation.

Task execution time (*T*_*ET*_) of an offloaded task is calculated as the time when a node has an executable task till the time when the task is executed. If *P*_*B*,*N*_ is the propagation delay between the task offloading node and the load balancer, and *T*_*WT*_ is the waiting time of a node at the load balancer, *P*_*B*,*F*_ is the propagation delay between the load balancer and fog computing node, *T*_*QD*_ is the queuing delay at fog node and *T*_*ET*_ is the executing time. Then task execution time of node *N* ($T_{N}^{ET}$) is calculated as:

$$\begin{array}{c} T_{N}^{ET}=P_{B,N}+T_{WT}+P_{B,F}+T_{QD}+T_{ET} \end{array}$$ (1)

If there are *K* offloading tasks initiated by IoT nodes in a specific time frame *T*_*i*_, then the accumulated delay ($T_{Acc}^{ET}$) of all the offloaded tasks by fog nodes in *J* number of time frames is calculated as:

$$\begin{array}{c} T_{Acc}^{ET}=\sum_{i=1}^{k}T_{NJ}^{ET} \end{array}$$ (2)

The purpose of a load balancer is to balance the offloaded tasks to minimize the execution time.

### Load balancer

*EOTE* − *FSC* introduced a load balancer, that acts as a bridge between fog nodes and IoT sensors. All the offloaded tasks from sensor nodes are forwarded to their directly connected load balancer that is backwardly connected with multiple fog nodes as illustrated in Fig 3. Each load balancer has real-time load information of all of its connected fog nodes. This helps in allocating the new offloaded tasks on one of its attached fog nodes by applying a load-balancing algorithm as described in the following section.

### Load balancing algorithm

For efficient execution of the offloaded task in the fog node, we introduced a load balancer for fog nodes in smart city applications. An efficient load balancing mechanism by modifying the state-of-the-art Greedy algorithm. This algorithm works the same as the greedy algorithm. However, it schedules all the tasks in descending order before assigning them to the fog nodes.

Suppose there are *n* tasks, that are required to be assigned to *m* number of fog computing nodes uniformly. The proposed scheme assigns tasks to each fog computing node in such a way that the next task will be assigned to the machine that holds the lowest load.

The greedy algorithm not only simply assigns tasks to each fog node but also considers the load on each fog node. Suppose, there are *m* identical machines; let us say that *n* tasks have processing time *t*_*i*_. Suppose we have three fog nodes. The first task goes to the first fog node, 2^*nd*^ task goes to the second fog node, and 3^*rd*^ task goes to the third fog node. When the 4^*th*^ task arrives, the greedy algorithm keeps track of all the tasks. It checks the load on each fog node and will assign the 4^*th*^ task to the fog node that has a lesser load. Assign the next task to the machine with the lowest load so far. This procedure will continue until all the tasks are completed. The maximum load on every machine is reduced. Rearrange all the tasks in the descending order [“*j*4”, 10], [“*j*2”, 8] [“*j*1”, 6], [“*j*7”, 6], [“*j*3”, 4], [“*j*5”, 2], [“*j*6”, 1].

After completing all these tasks using the modified greedy technique, the maximum load will be 13 seconds as fog node 1 has a load of 13, fog node 2 has a load of 12, and fog node 3 has a load of 12. However, it was 14 through a greedy algorithm.

The time complexity of our proposed load balancing algorithm is calculated as O(n) and can solve large-scale problems. The proposed algorithm for the load balancing node is shown in Algorithm 1.

**Algorithm 1: Proposed Load Balancing Algorithm**

**Input**: Tasks = *n*, tasks processing times *t*_1_, *t*_2_, *t*_3_, …, *t*_*n*_, number of machines *m*_1_, *m*_2_, *m*_3_, …, *m*_*n*_

**Output**: Assignment of tasks to machines

1 Initialize sets: *tasks*(*M*_*i*_) = ∅ (assigned tasks to *M*_*i*_) and *Load*(*M*_*i*_) = ∅ (total load of machine *M*_*i*_)

2 Sort the tasks in descending order according to their processing times

3 **for** *i* = 1 to *n* **do**

4  Assign the task *i* to the machine *M*_*k*_ with the smallest load

5  *tasks*(*M*_*k*_) = *tasks*(*M*_*k*_) ∪ {*i*}   // Assign the task to machine *M*_*k*_

6  *Load*(*M*_*k*_) = *Load*(*M*_*k*_) + *t*_*i*_   // Update the load of machine *M*_*k*_

7 **end**

8 Sort the machines by their load in ascending order

9 **for** *i* = 1 to *n* **do**

10  Sort the tasks assigned to each machine in non-increasing order of their processing times

11 **end**

12 **return** *tasks*

### Task execution with deadline

Fog nodes after receiving all the offloaded tasks are required to execute them. These received tasks are different in their sizes with varying sensitivity requirements as some of these tasks have high priority as compared to others and need to be executed on a priority basis. Sensitive tasks have to meet strict deadlines as compared to normal tasks and if a task cannot be executed before its deadline then it is useless. Each fog node has a certain task processing time and sometimes it is not possible to execute all tasks within their stipulated time. This requires optimal decision-making to perform those tasks within the task processing capacity of the fog node.

*EOTE* − *FSC* offers an efficient algorithm for fog nodes to execute the tasks by considering their deadlines. *EOTE* − *FSC* meets the following two main requirements of offloaded tasks.

- Tasks have different deadlines
- Tasks require different execution time

Suppose there are 9 tasks (J1 to J9) and they are divided into three different priority levels, with 100, 60 and 30 as most sensitive, medium sensitivity and low sensitivity levels respectively. These tasks have different deadlines and require different execution times represented in time slots as shown in Table 2.

**Table 2:** Tasks with varying sizes.

*Tasks*	*Priority*	*Deadline*	*Slots*
*J1*	*100*	*5*	*1*
*J4*	*100*	*6*	*2*
*J8*	*100*	*7*	*2*
*J3*	*60*	*2*	*1*
*J5*	*60*	*2*	*2*
*J9*	*60*	*1*	*1*
*J2*	*30*	*4*	*1*
*J6*	*30*	*5*	*2*
*J7*	*30*	*1*	*1*

The proposed algorithm creates a Gantt chart to meet most of the deadlines of the scheme in the following steps.

1. Sort the offloaded tasks in decreasing order according to their priority.
2. Make the Gantt chart; the length of the Gantt chart will be as long as the maximum deadline of the highest priority task.
3. Take the tasks one by one as they are assigned. Create a Gantt chart for the work and place it as far away from 0 as feasible to ensure it is done before the deadline.
4. Fill in the time slots that are required to execute the offloaded task.

**Fig 2:**
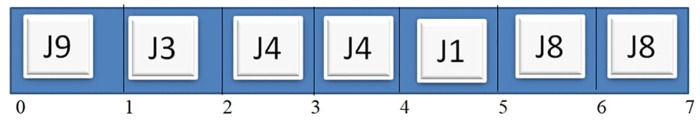
Assigning different sizes of task.

Fig 2 shows the task processing preference to achieve the deadlines of most of the tasks received by the fog computing node. As *J*8 task requires 2 slot sizes, and its deadline is 7. So this algorithm will check before the deadline 7 whether two consecutive slots are available or not. If available, it executes the task; otherwise, it will discard it. This procedure will be continued for all the tasks.

The time complexity of the task sequencing algorithm is calculated as *O*(*n*^2^). Due to its time complexity, the fog node applies this algorithm after a short interval of time to determine whether it can execute the offloaded tasks within time; otherwise, it discards it. A complete procedure of the task execution with deadlines is shown in Algorithm 2.

**Algorithm 2**: Enhanced task Sequencing with Deadlines

1 **Input:** *n* = Number of Tasks

2 *TD* = Task deadline

3 *TP* = Task Priority

4 *TS* = Task slots

5 *T*_*i*_ = current task

6 *D*_max_ = 0

7 **for** *i* = 1 to *n* **do**

8  Sort the tasks in decreasing order according to task priority

9  find the task that has maximum deadline (*TD*_max_)

10  *D*_max_ = *TD*_max_

11 **end**

12 *ET* = 0

13 *k* = *D*_max_

14 **for** *i* = 1 to *D*_max_ **do**

15  empty all slots

16 **end**

17 **for** *i* = 1 to *n* **do**

18  fill the last slot with *TD*_*i*_

19  *ET* = *ET* + 1

20  *k* = *k* − *TS*_*i*_

21  **if** *k* = 0 **then**

22  Stop the process

23  **end**

24 **end**

25 **Value of *ET* provides the total number of tasks selected for execution**

## System model and results

In this section, the performance of the proposed *EOTE* − *FSC* scheme will be analyzed in different prospects with state-of-the-art schemes. Before analyzing its performance, the system model along with simulation parameters is discussed.

### System model

In this work, we consider three smart city applications: smart health care, IoT-based home security, and industrial automation. Sensor nodes deployed for each application are supposed to have three different sensitivity levels with different deadline requirements, such as most sensitive, medium and low sensitivities. The deadline for similar sensitivity-level tasks is not fixed and varies according to their application requirements. In addition, the size and complexity level of these tasks vary and require different execution times.

**Fig 3:**
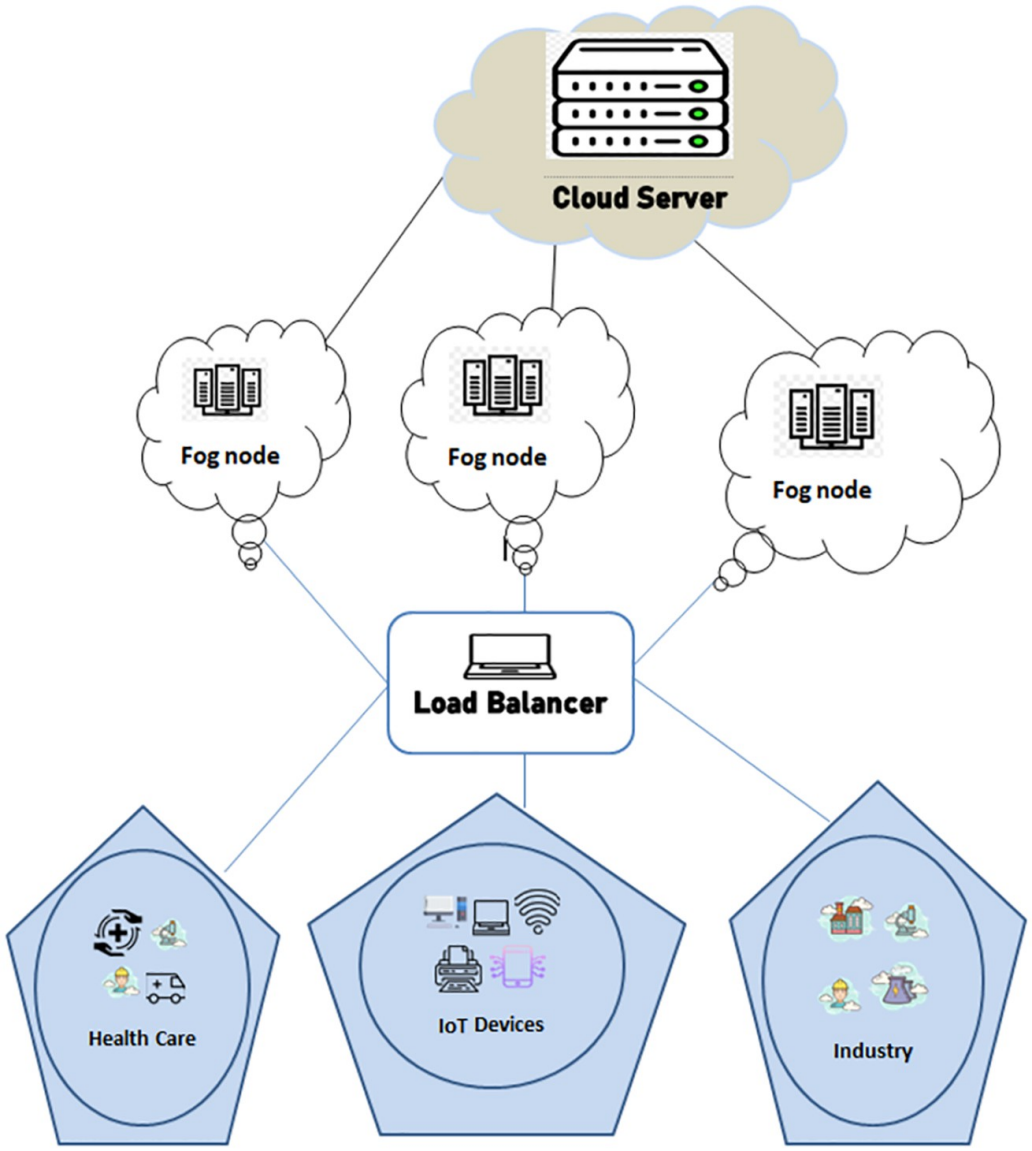
System model.

These IoT nodes of smart city applications are sparsely placed and are supposed to offload some of their tasks for timely execution. These tasks are required to be executed from the fog nodes that are placed in different vicinity of smart cities. Load balancing nodes are also located in different locations in such a way that each task offloading node is in direct connection with the load balancing nodes and a load balancer is backwardly connected with multiple fog computing nodes which are connected with the cloud at their back-end as shown in Fig 3. It is supposed that load balancing nodes and fog computing nodes have enough caching capacity to cache the offloaded tasks and none of the offloaded tasks is wasted due to overflow. The load balancing node assigns offloaded tasks to fog computing nodes by applying the proposed load balancing algorithm. A load-balancing node is supposed to have online knowledge about offloaded tasks present in the queuing lists of all of their directly connected fog nodes. All fog nodes are backwardly connected with the cloud.

In this work, *M* fog computing nodes are sparsely deployed in different areas of smart city in such a way that *M*_*i*_ fog node is associated with *N* number of smart city application networks. Sensor nodes are deployed in each network and offload their tasks to fog computing nodes. In *N*_*j*_ smart city application network, sensor nodes offload their tasks that are categorized as *t*_1_,*t*_2_ and *t*_3_ with low to high priority. If there are *V* fog nodes deployed to cover the specific area of the smart city, then the total number (*T*) of offloaded tasks received by all fog computing nodes is calculated as:

$$\begin{array}{c} T=\sum_{i=1}^{V}\sum_{j=1}^{Y}M_{i}\left(t_{j}\right) \end{array}$$ (3)

A load balancer is placed between sensor nodes and fog node and signals to noise ratio between the load balancer and sensor nodes (*γ*_*n*,*b*_) in a wireless channel is considered, the data rate (*DR*_1_) between the load balancer and all its connected nodes is calculated as

$$\begin{array}{c} DR_{1}=log_{2}\left(1+\gamma_{n,b}\right) \end{array}$$ (4)

Downloading data rate between the load balancer and its associated each of fog node (*DR*_2_) is calculated as:

$$\begin{array}{c} DR_{2}=log_{2}\left(1+\gamma_{b,f}\right) \end{array}$$ (5)

here *γ*_*b*,*f*_ is signal to noise ratio between the load balancer and fog node.

Let us have *m* indistinguishable fog node and *n* tasks. For each offloaded task *j*, the time required to process on one fog node is *t*_*j*_. Let *J*_*j*_ be the set of tasks assigned to the fog node. At any time instant, the overall execution time of fog node *a* is *F*_*a*_. The goal of the load balancing node is to minimize the execution time of all offloaded tasks on fog nodes, so that maximum execution time on any of the fog nodes in a network should be minimized.

### Simulation parameters

To evaluate the performance of our proposed *EOTE* − *FSC* scheme, a simulation environment following the system model is created in MATLAB simulator. Three different priority levels of tasks are taken from each of the three smart city applications with different sizes and varying time-binding requirements. The offloaded tasks are directly sent to the load balancing node that is directly connected with 2 to 7 fog nodes. To evaluate the performance of the fog node to meet different task deadlines, the offloaded tasks are uniformly divided into three categories. If there are 6 tasks assigned to fog nodes, then two tasks of high priority, two of medium priority and two for least priority tasks. A detailed list of simulation parameters is shown in Table 3.

**Table 3:** Simulation parameters.

Parameter	Value
Coverage area of load balancing node	200 m
Distance between fog nodes and load balancing nodes	50-150
Number of simultaneous offloaded tasks for each fog node	5-30
Number of fog nodes	2-7
Number of high priority tasks	2-8
Number of medium priority tasks	2-8
Number of low priority tasks	2-8
Time required for task execution (slots)	1-2
Slot duration (msec)	6
Tasks sizes (kB)	5-15
Processing of each fog node (Mb/s)	8

### Comparative results of proposed scheme

In this section, the performance of load balancing and task execution with deadlines of *EOTE* − *FSC* are evaluated in different prospects and with state-of-the-art algorithms. The performance of the proposed load balancing algorithm is compared with Round Robin, Greedy, and Round Robin with LJF algorithms in terms of the task execution time of the fog node. However, the performance of the task execution algorithm of our proposed *EOTE* − *FSC* for the fog node is compared with the First Come First Serve (FCFS) algorithm. The energy consumption in IoT is a crucial parameter. Transmitting offloaded tasks to the load balancer and receiving executed tasks consumes more energy, compared to executing tasks by an IoT node. However, executing tasks by IoT nodes is time-consuming as they have limited computing power compared to the fog node. In this study, our focus is to execute tasks within their deadlines and only consider the delays. We have not taken into account the energy constraints of IoT nodes.

#### Comparative performance analysis of load-balancing algorithm

The offloaded tasks received by the load balancer are required to be distributed among its attached fog nodes. The performance of the load-balancing algorithm is compared with above mentioned three other schemes. The results are analysed for varying number of arrived tasks with a fixed number (3) of fog nodes, and for varying number of fog nodes for a fixed number (24) of offloaded tasks with varying computing time.

**Fig 4:**
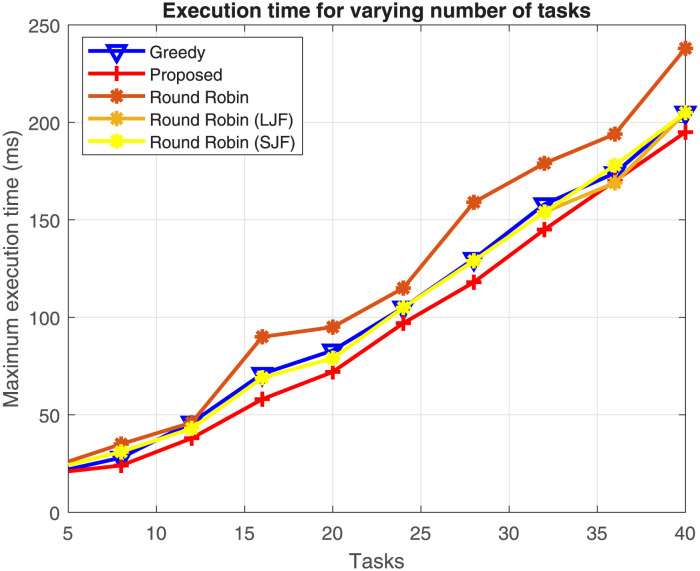
Task execution time with varying number of uploaded tasks.

Results shown in Fig 4 verify that the task execution time increases, when the number of offloaded tasks increases from 4 to 40 in different instances of times. These offloaded tasks are required to be executed by a fixed number of 3 fog nodes. The results further verify that the proposed scheme efficiently allocates the offloaded tasks on fog nodes resulting in reduced execution time as compared to the other four schemes. The results show that the proposed scheme executes offloaded tasks 29%, 21%, 15.5%, and 16.4% faster as compared to round-robin, greedy, round-robin with the LJF, and round-robin with the SJF algorithms respectively.

**Fig 5:**
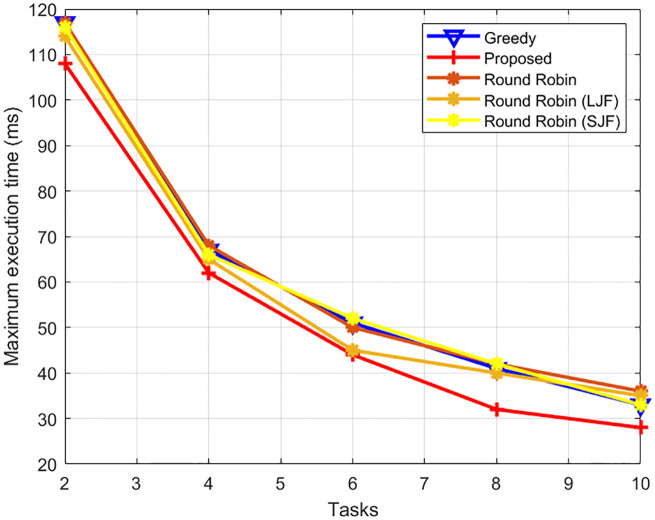
Task execution time with varying fog computing nodes.

The results shown in Fig 5 illustrate the total processing time when the number of fog nodes is increased from 2 to 10 and the total number of tasks is 24. The results show that the maximum task execution time of these offloaded tasks on one of the fog computing nodes reduces with the increase in the number of fog computing nodes as the same number of tasks are distributed on multiple fog nodes. The results further show that the execution time of our proposed load balancing scheme efficiently allocates nodes on its attached fog computing nodes as compared to the other four algorithms. The results show that the proposed scheme reduces the task execution time up to 28.5%, 27.3%, 23% and 24.4% as compared to round-robin, greedy, round-robin with LJF and round-robin with SJF respectively.

To overview the performance of our proposed scheme with the other four algorithms, the standard deviation is calculated to evaluate the deviation of these schemes from the mean value. The standard deviation (SD) is calculated as:

$$\begin{array}{c} SD=\sqrt{\sum_{i=1}^{N}{\left(X-Y\right)}^{2}/N} \end{array}$$ (6)

here,

X = mean time to compute the offloaded task

Y = time to execute the task by an algorithm

N = Number of instances

**Fig 6:**
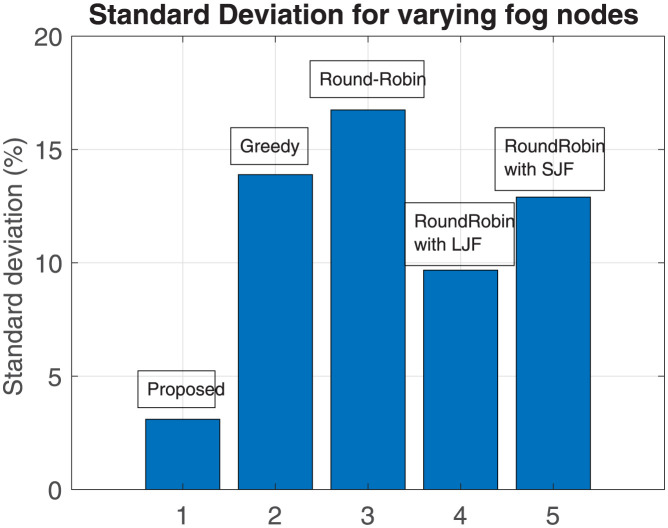
Standard deviation for varying fog nodes.

**Fig 7:**
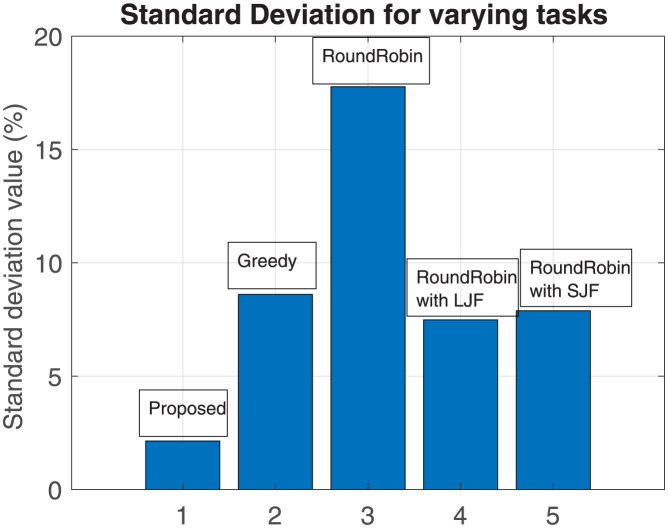
Standard deviation for a varying number of tasks.

Results shown in Figs 6 and 7 represent standard deviation values calculated for each algorithm for varying numbers of fog nodes and for varying numbers of offloaded tasks respectively. It is evident from the results that the deviation of the proposed scheme from the mean value is minimal as compared to the other three schemes and it remains close to the mean value.

#### Performance analysis of offloaded task execution algorithm

Fog nodes after receiving all offloaded tasks are required to execute. The task execution performance of our proposed algorithm is compared with the state-of-the-art *FCFS* algorithm for varying numbers of different priority levels of offloaded tasks.

**Fig 8:**
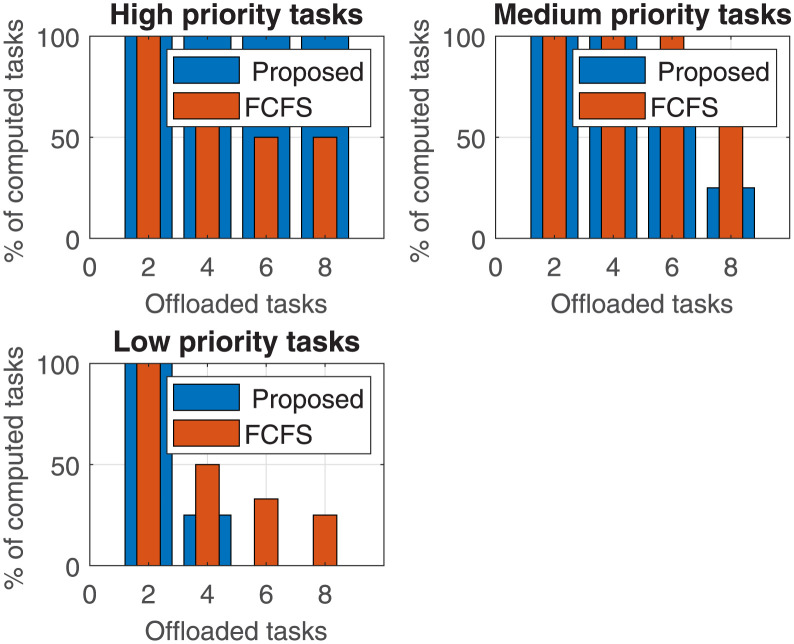
Percentage of tasks executed by a fog node for a varying number of offloaded tasks.

The percentage of tasks executed concerning task requests for three different priority tasks is calculated in Fig 8. Results include three sub-plots that represent three different priority level tasks: high-priority tasks, medium-priority tasks, and low-priority tasks. It is evident from the results that our proposed algorithm executed 100% high-priority tasks. When we compared our proposed algorithm with the FCFS algorithm, our proposed algorithm yielded up to 50% better. The percentage of task execution for ‘medium priority tasks’ is the same for up to 4 tasks. However, for 6 and 8 offloaded tasks, the FCFS scheme executes more tasks than the proposed scheme, because proposed scheme has already executed most of the highest priority tasks within its task execution capacity. However, when we had two tasks to execute, both schemes executed the same number of tasks in all priority tasks as all the tasks are within the processing capacity of the fog node.

**Fig 9:**
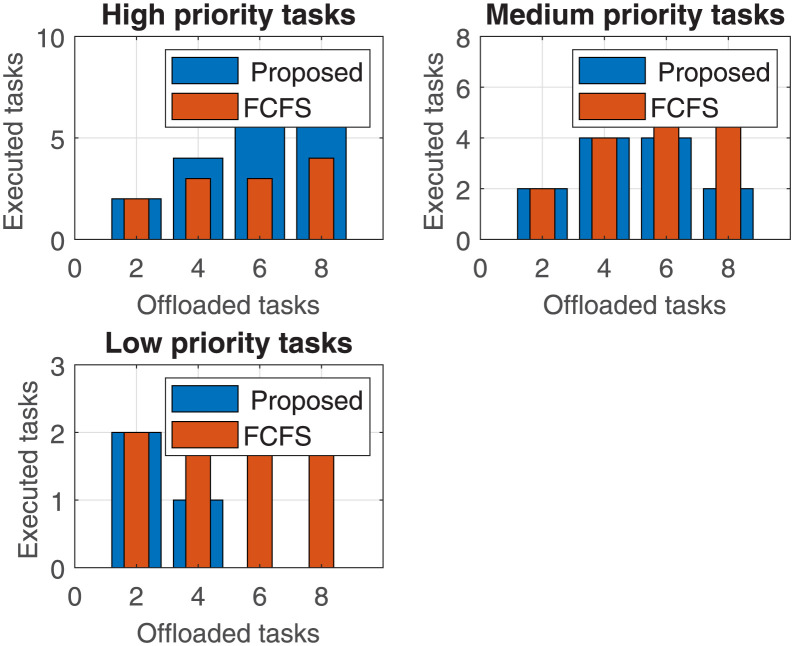
Total number of executed tasks for a varying number of offloaded tasks.

For better comparative analysis, the number of offloaded tasks executed against varying number of offloaded tasks for three different priority tasks is calculated in Fig 9. The figure includes three sub-figures that represent three priority-level tasks: high-priority, medium-priority, and low-priority tasks. It is evident from the results that our proposed algorithm executed up to 8 tasks in case of ‘high priority tasks’. When we compared our proposed algorithm with the FCFS algorithm, the FCFS algorithm executed half the number of tasks as compared to our proposed scheme. The number of tasks executed for ‘medium priority tasks’ is identical for both schemes, when the number of offloaded tasks is up to 4. However, when the number of offloaded tasks increased to 6 and 8 tasks, the FCFS scheme executed more tasks than the proposed scheme. This happens because the proposed scheme preferred to execute higher-priority tasks first and could not spare the processing capacity left to execute any low-priority tasks.

**Fig 10:**
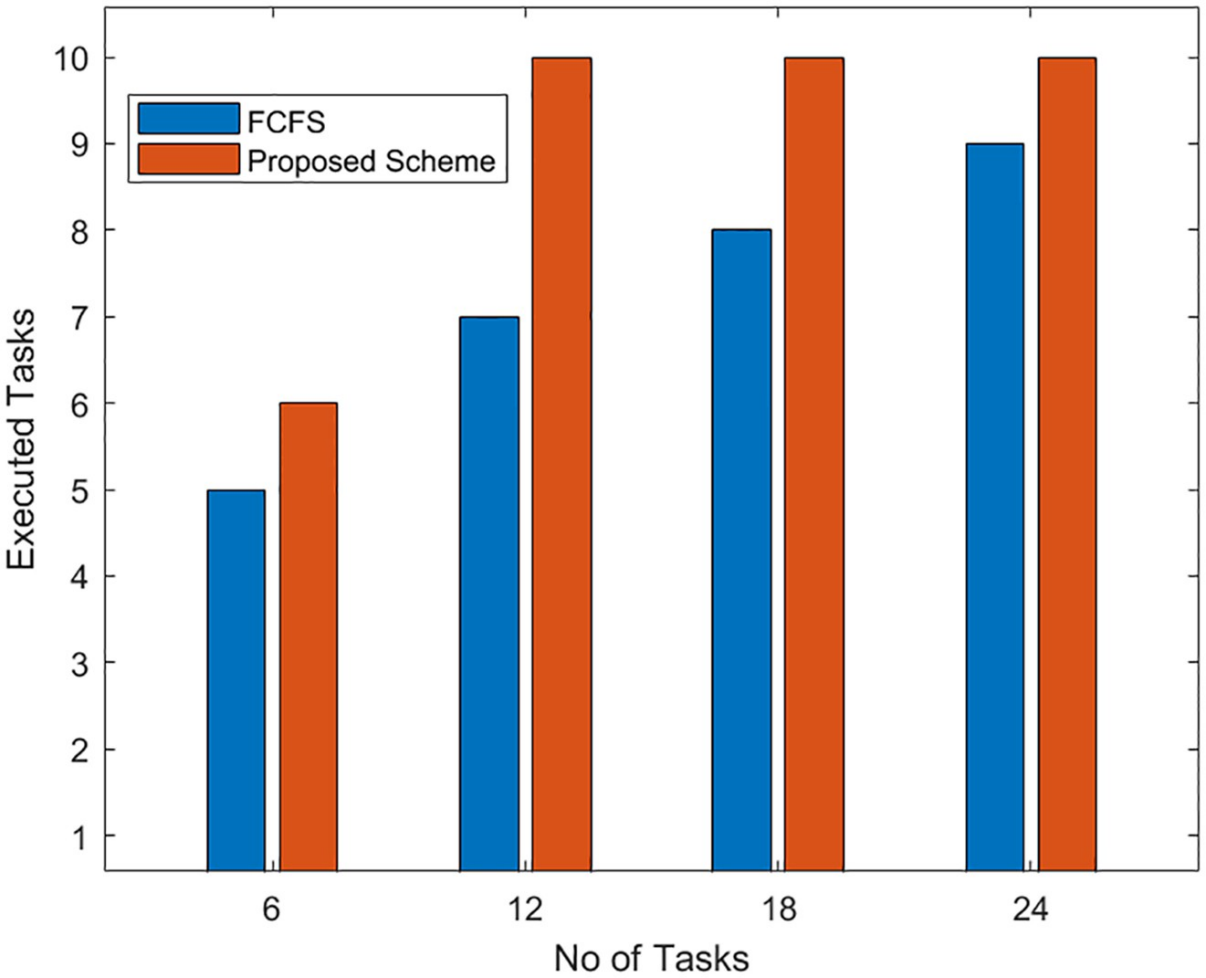
Task execution within deadline with same priority of offloaded.

The results shown in Fig 10 are observed when all the offloaded tasks have the same priority levels with varying deadlines and execution times. The results are observed for varying numbers of offloaded tasks to the fog node when it is processed for every 16 processing slot duration. In these results, the offloaded tasks are assumed to be executed by the fog node in an even number of processing slots. It is evident from the results that the proposed *EOTE* − *FSC* allows the fog node to execute a higher number of the offloaded tasks within their deadline. The results further show that the proposed algorithm could not execute more than 10 tasks when the number of offloaded tasks increases from 12. This is due to its processing limit as all 16 processing cycles have been allocated to tasks and no more tasks within their deadline can be executed. On the other hand, offloaded tasks selected in FCFS keep on increasing with the increase in offloaded tasks but they remain less from the proposed scheme.

## Conclusion

In this work, an efficient offloaded tasks execution scheme for fog-enabled smart city applications *EOTE* − *FSC* is proposed. The *EOTE* − *FSC* introduced a concept of load-balancing nodes and proposed load balancing and task execution with deadline algorithms. The load balancing results were compared with round-robin, Greedy, round-robin with LJF and round-robin with SJF algorithms. The results show that our proposed scheme reduced the processing time by up to 29%, 27.3%, 23%, and 24.4% against round-robin, greedy, round-robin with the LJF, and round-robin with the SJF algorithms respectively. Task execution with the deadline algorithm was compared with the FCFS algorithm. The proposed scheme outperformed FCFS in terms of highest-priority tasks by compromising low-priority tasks. The majority of the highest priority tasks are executed and most of the medium priority tasks are also executed before meeting their deadline.

Several smart city applications require mobile nodes, so a load-balancing mechanism by considering the mobility of these nodes needs to be considered in future studies.
